# Phase-change perfluorocarbon nanodroplets enable spatiotemporal control of ultrasound and photoacoustic imaging and therapy

**DOI:** 10.1007/s13534-026-00587-8

**Published:** 2026-06-01

**Authors:** Euisuk Chung, Robert J. Nikolai, Seoyoon Song, Stanislav Y. Emelianov

**Affiliations:** 1 School of Electrical and Computer Engineering, Georgia Institute of Technology, Atlanta, GA 30332 USA; 2 Wallace H. Coulter Department of Biomedical Engineering, Georgia Institute of Technology and Emory University School of Medicine, Atlanta, GA 30332 USA

**Keywords:** Perfluorocarbon nanodroplets, Contrast agents, Ultrasound imaging, Photoacoustic imaging, Acoustic droplet vaporization, Optical droplet vaporization

## Abstract

Phase-change perfluorocarbon nanodroplets (PFCnDs) are submicrometer, liquid-core agents capable of generating strong ultrasound (US) and photoacoustic contrast following external stimulation. This selective vaporization allows site-specific signal generation and facilitates imaging beyond the vascular compartment. The behavior of PFCnDs is determined by a thermodynamic landscape in which Laplace pressure and nucleation kinetics jointly influence vaporization thresholds and post-vaporization outcomes, including persistent gas bubble formation or rapid recondensation. This review summarizes the mechanistic framework underlying acoustic droplet vaporization, optical droplet vaporization, and combined optical-acoustic droplet vaporization of PFCnDs. These phase-change mechanisms are discussed in relation to formulation variables such as perfluorocarbon species, size distribution, shell mechanics, and absorber loading, as well as exposure conditions including acoustic pressure, frequency, pulse structure, and optical fluence. The review further examines how these coupled parameters govern vaporization dynamics, imaging persistence, and associated bioeffects. Imaging applications, including contrast-enhanced US, extravascular and lymphatic imaging, super-resolution, and multiplexed approaches that leverage vaporization–recondensation behavior, are surveyed. Therapeutic applications, such as drug delivery and barrier-opening techniques, are also examined alongside clinical translation priorities, including manufacturing, in vivo fate verification, reproducible vaporization, and standardized safety dosimetry.

## Introduction

**Fig. 1:**
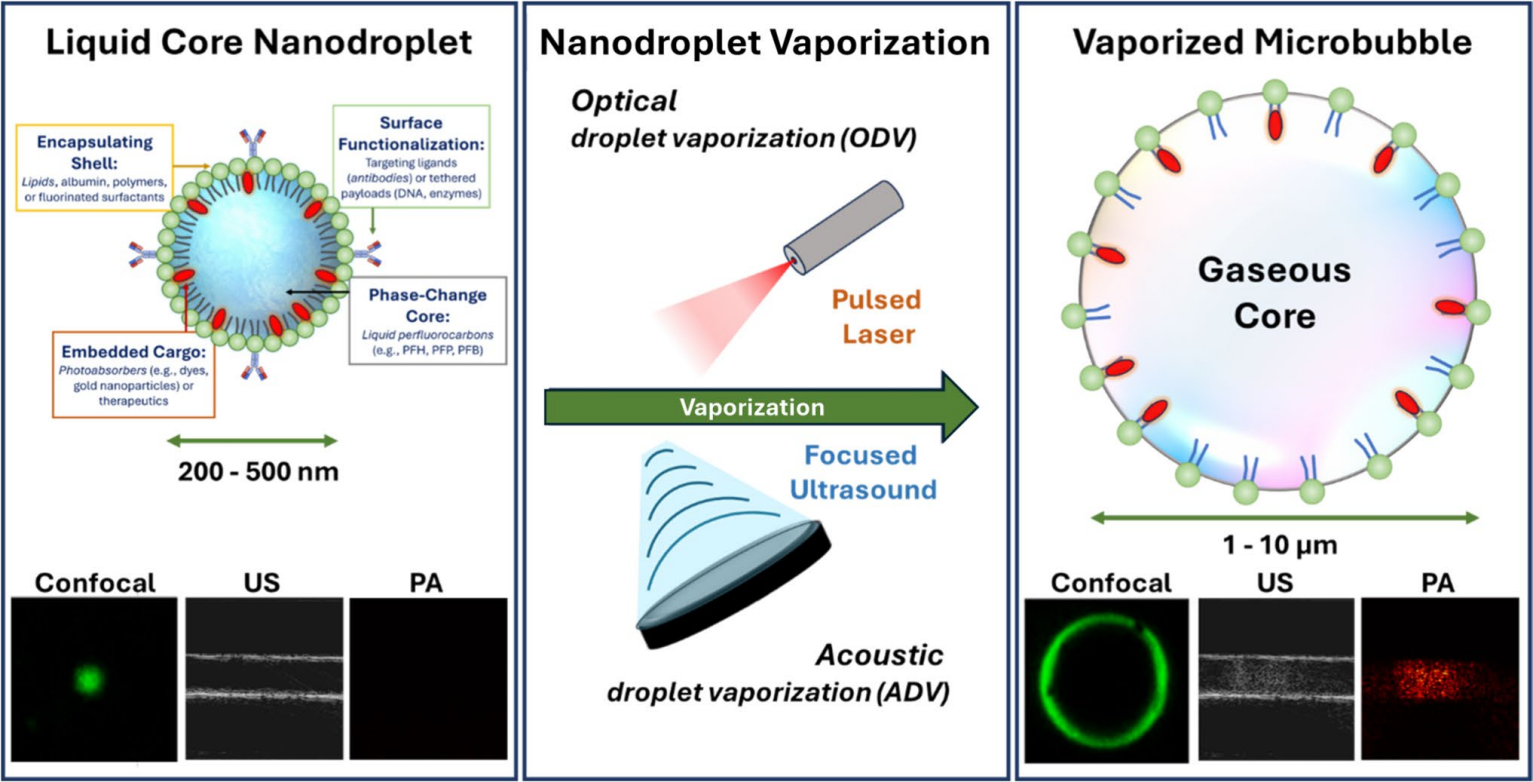
Schematic of phase-change perfluorocarbon nanodroplets and phase-change induced contrast-enhanced images. (Left) The initial state of nanodroplets (~ 200–500 nm) consists of a liquid perfluorocarbon core encapsulated by a stabilizing shell. Optical absorbers can be incorporated to enable optical triggering, and the shell surface can be functionalized with cargos or targeting moieties. A confocal fluorescence image illustrates the nanodroplet before vaporization. Ultrasound (US) and photoacoustic (PA) images show minimal baseline signal prior to vaporization. (Middle) Nanodroplets are vaporized via optical droplet vaporization (ODV) using laser irradiation or acoustic droplet vaporization (ADV) using ultrasound exposure. (Right) Upon triggering, the perfluorocarbon core transitions to a gaseous state, expanding to micron-scale bubbles (~ 1–10 μm) and producing enhanced US echogenicity and PA contrast, as illustrated by vaporized microbubble images. Confocal fluorescence microscope images are reproduced with permission from [26]

Phase-change perfluorocarbon nanodroplets (PFCnDs) are being actively developed as phase-change ultrasound (US) contrast agents that expand the functionality of conventional microbubble-based agents. The principal advantage of PFCnDs is their on-demand phase change upon external triggering [1–5]. Although microbubbles are widely used in clinical US imaging, their microscale restricts usage to intravascular applications, limiting access to extravascular targets [1, 2, 6]. Furthermore, their short circulation persistence, typically around 10 min, further restricts the practical imaging and treatment window [1, 3]. PFCnDs overcome these limitations by initially circulating as liquid-core droplets that transition to gas-core microbubbles only upon triggering [7–10]. In the initial liquid state, the perfluorocarbon core can be formulated as submicrometer droplets, generally in the range of hundreds of nanometers [7, 10]. This reduced size allows nanodroplets to access extravascular compartments and lymphatic pathways, and to persist in circulation for extended periods [7, 8, 11–14]. Upon exposure to external stimuli such as ultrasound or laser energy, a liquid–gas phase change can occur within the perfluorocarbon core (Fig. 1) [7, 15, 16]. The resulting transition expands the droplet into a gas-core microbubble that provides hyperechoic ultrasound contrast, with its diameter increasing several-fold depending on the formulation and vaporization conditions [7, 8, 17]. The switchable properties of PFCnDs facilitate site-selective contrast generation, offering improved spatial and temporal control at designated sites compared to single-mode contrast agents.

PFCnDs are composed of a perfluorocarbon core surrounded by a biocompatible shell, such as lipids, albumin, polymers, and fluorinated surfactants. Various fabrication and size-selection strategies, such as microbubble condensation, ultrasonic processing, or microfluidics, have been reported to generate submicrometer droplets suitable for biomedical applications [18–24]. The functional properties of PFCnDs can be tailored to specific biomedical applications by incorporating optical absorbers, engineering shells with targeting ligands, or loading therapeutics within the core or onto the shell [7, 25–30]. The flexibility of nanodroplet formulation enables a broad range of applications beyond conventional contrast-enhanced ultrasound (CEUS), encompassing various imaging modalities and therapeutic approaches that exploit triggered phase change and post-vaporization bubble dynamics [31]. Current research focuses on optimizing both synthesis parameters (e.g. composition, perfluorocarbon species, and droplet size distribution) and phase-change parameters (e.g. acoustic pressure/frequency/pulse structure and optical fluence) to address specific application and safety requirements [8, 10, 15, 32–34].

Previous reviews have introduced phase-change PFCnDs and their biomedical applications. As the field expands beyond acoustic triggering toward optical and combined optical-acoustic triggering, a comprehensive review is timely. Expanding upon this foundation, the present review offers an integrative perspective on phase-change PFCnDs. It highlights recent advances in acoustic, optical, and combined optical-acoustic droplet vaporization strategies within a unified framework that connects formulation parameters, vaporization behavior, and subsequent imaging and therapeutic performance. In addition to surveying established and emerging applications, this review emphasizes translational issues of growing importance, such as reproducible vaporization, in vivo fate, and safety considerations for future clinical use. The discussion begins with nanodroplet formulation and phase-change techniques, then addresses imaging and therapeutic strategies, and finally summarizes the current limitations for clinical application and potential paths toward translation.

## Composition and synthesis of perfluorocarbon nanodroplets

### Perfluorocarbon core

**Fig. 2:**
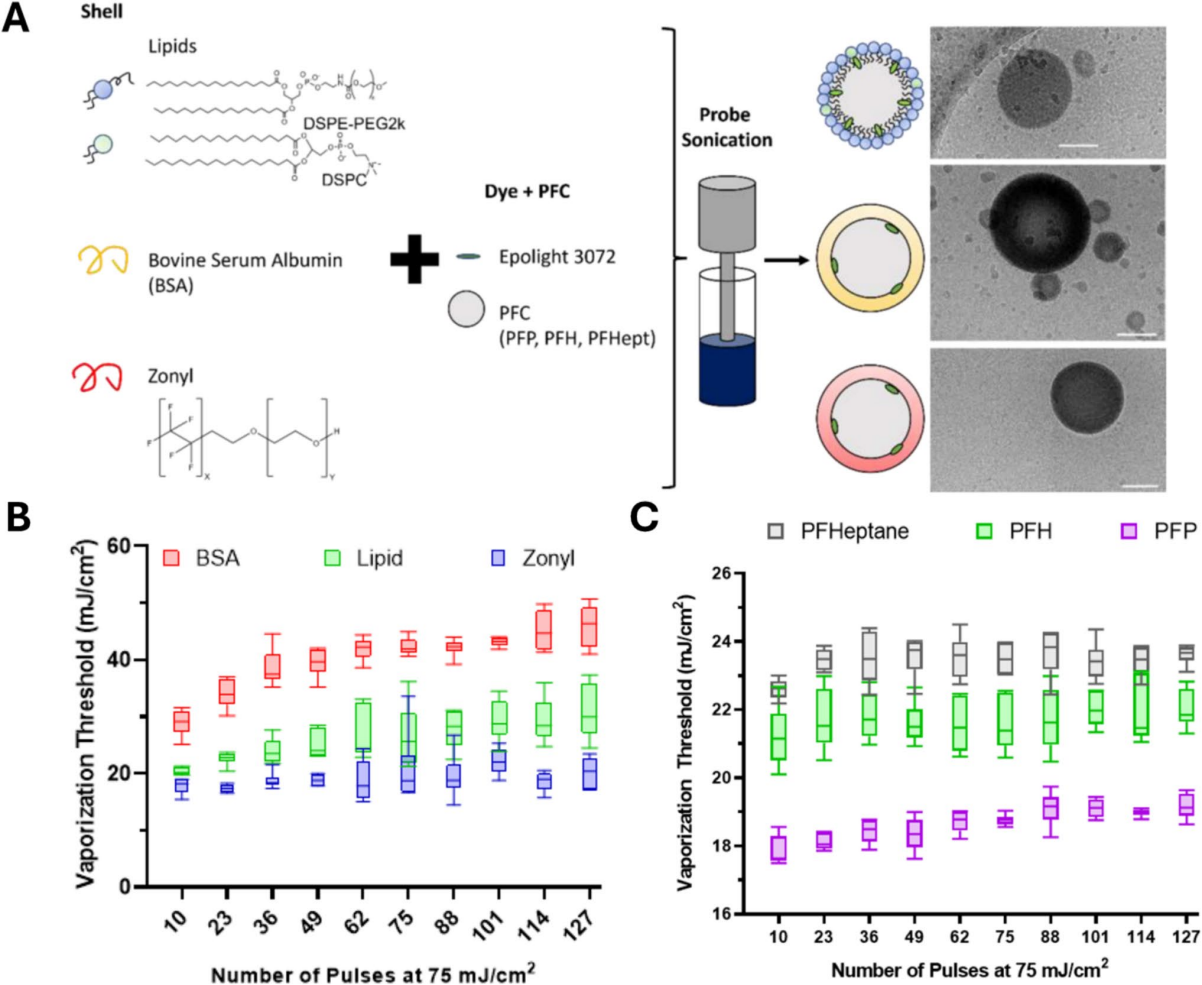
**A** The schematic illustrates the different shell components and different perfluorocarbons (PFC), along with cryogenic transmission electron microscopy images taken of each shell formulation with perfluorohexane (PFH). The scale bar represents 100 nm. **B** The vaporization threshold of PFHnDs with different shells after exposure to varying numbers of laser pulses (75 mJ/cm^2^). **C** The vaporization thresholds of lipid shell PFCnDs with different cores after exposure to varying numbers of laser pulses (75 mJ/cm^2^). Reproduced with permission from [17]

The perfluorocarbon (PFC) core determines volatility, storage stability, and the phase-change operating window. Low-boiling-point PFCs, such as perfluorobutane (PFB) and octafluoropropane (OFP), reduce vaporization thresholds but often exhibit reduced stability and an increased risk of spontaneous vaporization (Fig. 2C) [35, 36]. In contrast, high-boiling-point PFCs, including perfluorooctane (PFO) and perfluorohexane (PFH), enhance stability but require higher acoustic or optical thresholds and enable controlled vaporization dynamics, such as repeated vaporization-recondensation cycles that facilitate advanced imaging strategies like blinking-based super-resolution [37, 38]. Core selection is therefore determined by the required balance between stability and vaporization efficiency and is reported alongside droplet size and shell type to contextualize threshold measurements [9, 15, 35, 36, 38–41].

### Shell composition and functionalization

The shell stabilizes the PFC core against coalescence and provides a platform for functionalization. Lipid-based shells are the most widely used formulation, typically composed of phospholipids such as 1,2-distearoyl-sn-glycero-3-phosphocholine (DSPC) or 1,2-dipalmitoyl-sn-glycero-3-phosphocholine (DPPC), often combined with polyethylene glycol (PEG)-lipids to extend circulation time [8, 24]. Lipid shells exhibit lower interfacial rigidity than polymer-based shells, resulting in lower vaporization thresholds, making PFCnDs suitable for applications requiring sensitive triggering [9]. Shell composition directly affects nanodroplet mechanical properties: increasing PEG-lipid content reduces surface tension and lowers vaporization thresholds, whereas the addition of cholesterol or saturated lipids increases shell rigidity and stability [24].

Polymer-based shells, such as poly(lactic-co-glycolic acid) (PLGA) and poly(vinyl alcohol) (PVA), provide enhanced mechanical stability and controlled drug release capabilities [4, 42]. PLGA shells facilitate encapsulation of hydrophobic therapeutics within the polymer matrix and support sustained release upon triggering. Albumin-based shells offer biocompatibility and endogenous biodegradability, but exhibit higher vaporization thresholds due to increased interfacial tension (Fig. 2B) [8]. Fluorinated surfactants reduce interfacial tension between the PFC core and aqueous phase, lowering vaporization requirements and improving formulation stability [9].

Functionalization enables targeting and multimodal imaging. Optical absorbers, such as indocyanine green (ICG), porphyrins, or plasmonic gold nanoparticles, are incorporated into the shell to enable optical droplet vaporization and photoacoustic imaging [25, 26, 43]. The absorber concentration must be optimized to balance photothermal efficiency against potential toxicity and changes to shell mechanics. Targeting ligands, such as antibodies, peptides, or aptamers, can be conjugated to the shell surface via PEG linkers or maleimide chemistry to enable active targeting of molecular biomarkers, including VEGFR2, EGFR, or integrins [29, 30, 44]. The spatial arrangement and density of targeting moieties influence binding affinity and circulation pharmacokinetics, requiring careful optimization for each application.

### Synthesis method

Fabrication of PFCnDs requires precise control over size distribution, PFC loading, and formulation stability. Multiple synthesis approaches have been developed, each offering specific advantages and limitations. Microbubble condensation generates PFC-filled microbubbles via conventional agitation or sonication, followed by controlled cooling or pressurization to induce gas-to-liquid condensation [20, 23]. This approach produces nanodroplets with diameters ranging from 200 to 500 nm, though the resulting size distribution is often polydisperse. The condensation process requires careful temperature control to prevent incomplete phase change or re-expansion during storage.

Ultrasonic emulsification utilizes tip sonication to disperse the PFC phase into an aqueous solution containing dissolved shell materials [20]. Acoustic cavitation and shear forces fragment the PFC into submicrometer droplets, which are rapidly stabilized by shell adsorption. Sonication amplitude and duration directly affect the size distribution. Tip sonication generally produces polydisperse populations with coefficients of variation exceeding 20%, limiting reproducibility for applications requiring narrow size distributions [20]. Post-synthesis size selection via differential centrifugation or flotation can narrow the distribution, though at a reduced yield.

Microfluidic synthesis offers superior control over droplet size, PFC loading, and reproducibility [10, 21, 22]. Flow-focusing or T-junction geometries enable continuous generation of monodisperse droplets by precisely controlling flow rates and channel dimensions. Microfluidic platforms have achieved coefficients of variation below 5%, substantially improving the reproducibility of vaporization thresholds compared to bulk emulsification methods [10].

Post-synthesis processing typically includes purification steps to remove excess shell material, unencapsulated PFC, and suboptimal size fractions. Centrifugation-based washing separates nanodroplets from free lipids and soluble excipients, though repeated centrifugation can induce aggregation or phase change in volatile formulations [20]. Tangential flow filtration (TFF) offers a gentler, scalable alternative that continuously removes contaminants while concentrating the nanodroplet suspension [22].

### Characterization and quality control

Comprehensive physicochemical characterization is essential to ensure reproducibility and predict in vivo behavior. Size distribution is typically measured via dynamic light scattering (DLS), nanoparticle tracking analysis (NTA), or microscopy [8, 20]. DLS provides rapid ensemble measurements but can overestimate size in polydisperse samples, while NTA enables single-particle resolution and direct visualization of size distributions. Electron microscopy confirms morphology and shell thickness and reveals structural details not accessible to optical methods (Fig. 2A). Zeta potential measurements assess surface charge, which influences circulation stability and biological interactions. PEGylated formulations typically exhibit near-neutral zeta potentials (− 10 to 10 mV), minimizing protein adsorption and opsonization [10].

PFC content and encapsulation efficiency can be quantified via gas chromatography, fluorine nuclear magnetic resonance (19F-NMR), or gravimetric analysis after shell removal [8]. These measurements verify complete core loading and detect potential phase separation or PFC loss during storage. Stability assessments such as size tracking, PFC retention, and vaporization threshold monitoring over time are critical for shelf-life determination and storage condition optimization [8, 24].

## Vaporization modalities of phase-change perfluorocarbon nanodroplets

The liquid state of the PFC core in nanodroplets is determined by the combined effects of the bulk thermodynamic properties of the perfluorocarbon and the pressure increase generated at the curved droplet interface. Depending on the PFC species, the boiling point of the core at atmospheric pressure may be either above or below body temperature (~ 36 °C). Even so, droplets containing compounds such as perfluoropentane (PFP; boiling point, 29 °C) or perfluorohexane (PFH; boiling point, 56 °C) can remain liquid because the curved interface elevates the internal pressure according to Laplace’s law (ΔP = 2γ/R). This pressure elevation increases the effective boiling point of the core, thereby stabilizing the liquid phase. However, under external stimulation, vaporization can occur when the nucleation barrier is sufficiently reduced or when local pressure–temperature conditions are transiently shifted. Local pressure reduction during acoustic rarefaction or core heating induced by photothermal absorption can facilitate vapor embryo formation and subsequent bubble growth [7, 8, 15, 45]. The vaporization response depends on intrinsic formulation parameters, such as PFC species, droplet size distribution, and interfacial properties, as well as extrinsic exposure parameters, including peak negative pressure (PNP), frequency, and pulse sequence for acoustic vaporization [9, 15]. For laser triggering, the key parameters are optical fluence, wavelength, and pulse duration [10].

Defining a specific vaporization threshold is complex because it is rarely a single-valued material property. Even in well-controlled formulations, vaporization typically follows a probabilistic distribution: droplets are polydisperse to some extent, local acoustic/optical fields are heterogeneous in tissue, and nucleation is stochastic. Various methods have been used to extract a single threshold value from this distribution. For example, some studies fit a sigmoid to the measured imaging signal as a function of acoustic pressure or optical fluence and extrapolate to estimate vaporization onset [17]. More broadly, vaporization is often characterized along four axes: (i) threshold or operating window, (ii) vaporization probability or fraction as a function of exposure, (iii) post-transition fate (persistent gas phase versus recondensation), and (iv) likelihood of collateral bioeffects (e.g., inertial cavitation, excessive heating). These four axes provide a consistent framework for comparing modalities independent of downstream applications [8].

Thermodynamic and kinetic analyses are critical for understanding PFCnD vaporization mechanisms because nanoscale effects render the perfluorocarbon’s bulk boiling point inadequate for the prediction of vaporization thresholds [7, 45]. For nanoscale droplets, internal pressure exceeds the ambient pressure by the Laplace pressure, a contribution that arises from surface tension and becomes significant at small radii. For spherical droplets, the pressure offset is given by ΔP = 2γ/R, where γ is the surface tension, and R is the nanodroplet radius [9]. At equilibrium, therefore, smaller droplets exhibit higher Laplace pressures, elevating their vaporization thresholds. However, equilibrium analysis alone does not fully account for the experimentally observed stability and vaporization threshold behavior, necessitating consideration of nucleation kinetics [15, 46, 47].

The homogeneous nucleation model indicates that vaporization requires overcoming a free-energy barrier to forming a critical vapor embryo. Spontaneous vaporization can occur near a spinodal-like limit at a significant fraction of the critical temperature, depending on the liquid and environmental conditions [23, 47, 48]. Vaporization triggering may lower the nucleation threshold through acoustic pressure rarefaction, direct heating, photothermal effects, or their combination.

Once triggered, PFCnDs rapidly expand into gaseous microbubbles. This process is not merely a reversible switch and results in outcomes including gas-phase persistence or recondensation [37]. These outcomes are determined by formulation and exposure conditions, which in turn influence imaging persistence, therapeutic efficacy, and safety.

From a practical design standpoint, PFCnD behavior represents a balance among vaporization sensitivity, post-vaporization fate, and the risk of collateral bioeffects. Lower-boiling-point PFC cores generally reduce vaporization thresholds but compromise storage stability, while higher-boiling-point cores require higher exposure conditions for vaporization yet offer greater formulation stability and, in some cases, enable repeated vaporization–recondensation cycles. Droplet size and shell mechanics also influence the operating window: smaller droplets and more rigid shells enhance stability but necessitate stronger vaporization conditions, whereas larger droplets and less rigid shells lower the vaporization threshold. In absorber-loaded formulations, increased absorber loading and appropriate spectral matching can lower optical vaporization thresholds; however, nonuniform loading can broaden threshold distributions, and excessive loading can increase the risk of unwanted thermal or mechanical bioeffects. Therefore, formulation design should be tailored to the intended application, such as low-threshold formulations for sensitive triggering, recondensing formulations for blinking-based imaging, or more persistent and cavitation-prone formulations for mechanically mediated therapeutic effects. Representative perfluorocarbon cores, their boiling points, storage stability, and reported ADV/ODV vaporization parameters are summarized in Table 1.

**Table 1:** Perfluorocarbon core selection: boiling point, vaporization parameters (ADV/ODV), and storage stability

PFC core	Boiling point (ºC)	Storage stability	Vaporization type	Vaporization parameter	References
Perfluorooctane (PFO; C_8_F_18_)	103–104	Excellent	ADV	4.5 MPa @ 2.5 MHz (single burst, 6-cycle pulse)	[60]
			ADV	1.5 MPa @ 1.1 MHz 3.5 MPa @ 2.5 MHz 6.7 MPa @ 3.3 MHz (12 µs duration)	[61]
Perfluorohexane (PFH; C_6_F_14_)	56	Good (Balanced stability)	ADV	4 MPa @ 1, 2.5, 9.4 MHz (single burst, 6-cycle pulse)	[60]
			ADV	11.8 MPa @ 1.1 MHz (single burst, 10-cycle pulse)	[62]
			ADV	2.1 MPa @ 1.1 MHz (duration: 7 min, repetition frequency: 11 kHz, 5 cycles per pulse)	[63]
			ODV	1.0 J/cm^2^ @ 1,064 nm (width: 700 ps, repetition frequency: 2 kHz)	[16]
			ODV	22 mJ/cm^2^ @ 1,064 nm (width: 5 ns, repetition frequency: 10 Hz)	[17]
			ODV	28 mJ/cm^2^ @ 1,064 nm (width: 4–7 ns, repetition frequency: 10 Hz)	[24]
			ODV	20–60 mJ/cm^2^ @ 1,064 nm (width: 5 ns, repetition frequency: 10 Hz)	[64]
			ODV	24 mJ/cm^2^ @ 1,064 nm (width: 5–7 ns, repetition frequency: 20 Hz)	[65]
Perfluoropentane (PFP; C_5_F_12_)	29	Moderate	ADV	6 MPa @ 5 MHz (single burst, 10-cycle pulse)	[35]
			ADV	2.3–3.5 MPa @ 10 MHz (single burst, 5–10 cycle pulse)	[51]
			ADV	4 MPa @ 1, 2.5, 9.4 MHz (single burst, 6-cycle pulse)	[60]
			ADV	11.8 MPa @ 1.1 MHz (single burst, 10-cycle pulse)	[62]
			ODV	0.8 J/cm^2^ @ 1,064 nm (width: 700 ps, repetition frequency: 2 kHz)	[16]
			ODV	19 mJ/cm^2^ @ 1,064 nm (width: 5 ns, repetition frequency: 10 Hz)	[17]
			ODV	22 mJ/cm^2^ @ 760 nm (width: 5–7 ns, repetition frequency: 20 Hz)	[65]
Perfluorobutane (PFB; C_4_F_10_)	− 2	Poor (risk of spontaneous vaporization)	ADV	0.45—0.6 MPa @ 1.5 MHz (duration: 5 min, repetition frequency: 5 Hz, 10 k cycles per pulse)	[27]
			ADV	2 MPa @ 8 MHz (single burst, 2-cycle pulse)	[39]
			ADV	2 MPa @ 1 MHz 3 MPa @ 5.5 MHz ~ 3.75 MPa @ 8 MHz (single burst, 2-cycle pulse)	[40]
			ADV	4 MPa @ 5 MHz (single burst, 10-cycle pulse)	[35]
Octafluoropropane (OFP; C_3_F_8_)	− 36	Very poor (high risk of spontaneous vaporization)	ADV	1.1 MPa @ 8 MHz (single burst, 2-cycle pulse)	[39]
			ADV	0.34 MPa @ 4 MHz (single burst, 20-cycle pulse)	[66]

Comparison of representative perfluorocarbon (PFC) cores used in phase-change droplet formulations, listing chemical formula and boiling point together with reported vaporization conditions for acoustic droplet vaporization (ADV; peak negative pressure range, ultrasound frequency, and pulse sequence) and, where available, optical droplet vaporization (ODV; laser fluence range and wavelength with beam width and repetition frequency). Qualitative storage stability is summarized for each core

### Acoustic droplet vaporization (ADV)

Acoustic droplet vaporization (ADV) induces liquid-to-gas conversion via ultrasound rarefaction, transiently lowering the nucleation threshold within the PFC core [7]. The ADV threshold is primarily determined by the PNP, but the experimentally observed threshold also depends strongly on frequency and pulse structure [15]. Longer tone bursts and higher pulse repetition frequency (PRF) reduce the exposure required to vaporize a given droplet population, consistent with the probabilistic nature of nucleation and the role of repeated attempts at embryo formation.

Droplet size is a critical factor: smaller droplets experience higher Laplace pressure as previously discussed, resulting in greater stability and requiring higher acoustic pressures [10, 49, 50]. PFC volatility also plays a key role; lower-boiling compounds vaporize at reduced pressures but may compromise storage stability [9, 35, 36, 38–41]. Shell composition further affects thresholds: thin lipid shells minimize interfacial rigidity and lower the PNP required for vaporization, whereas polymer shells increase mechanical stability but require increased vaporization energies [8].

Upon triggering, droplets rapidly expand to several times their original size, generating microbubbles that produce strong ultrasound backscatter [7]. Bubble persistence depends on ambient temperature, dissolved gas content, and shell properties [15, 37, 51]. These factors directly influence the duration of imaging contrast and therapeutic bio effects.

In ADV, both nanodroplet vaporization and imaging rely on ultrasound, which can lead to mutual interference [7]. Vaporization triggering pulses may appear as imaging artifacts, and imaging pulses may cause unintended vaporization. Furthermore, depending on formulation, ADV thresholds may exceed the FDA-approved mechanical index. Current research focuses on reducing the ADV threshold below safe exposure limits by optimizing droplet design and refining pulse sequences to maintain effective vaporization and high-quality imaging.

### Optical droplet vaporization (ODV)

Optical droplet vaporization (ODV) induces a phase change through the combined effects of photothermal heating and photoacoustic pressure generation within optical absorber-loaded nanodroplets [16, 25]. Upon pulsed laser irradiation, optical absorbers such as gold nanoparticles or near-infrared (NIR) dyes convert optical energy into highly localized transient heating near the absorber-PFC interface. The pulsed absorption also generates a photoacoustic pressure transient via thermoelastic expansion, which can assist nucleation by transiently modulating local pressure and reducing the effective nucleation barrier [16, 25, 26]. Although the bulk temperature rises during pulsed laser irradiation is modest (approximately 1 °C), the interfacial temperature near the optical absorber during nanosecond pulses can be substantially higher than the bulk average, enabling vapor embryo formation. Overall, ODV is promoted by the joint action of localized heating and the photoacoustic pressure transient, with their relative contributions depending on pulse duration, absorber loading, and incident fluence [17, 34]. The resulting vapor nucleates and expands into a microbubble, producing a photoacoustic signal and subsequent ultrasound contrast [25].

The ODV threshold primarily depends on optical fluence and varies significantly with PFC volatility: lower-boiling PFCs vaporize at lower fluences than higher-boiling PFCs [17, 35, 39]. As with ADV, droplet size influences the ODV threshold because of variations in Laplace pressure. Non-uniform uptake of the absorber by nanodroplets broadens the distribution of ODV thresholds, even in monodisperse nanodroplet populations [10]. Photothermal efficiency depends on absorber type, concentration, and spectral alignment with the laser wavelength [26, 43]. Optical absorbers tuned to the NIR region enable deeper tissue penetration and require lower vaporization fluence compared to visible-light absorbers [16].

ODV offers several advantages compared to ADV. Unlike ADV, ODV does not interfere with acoustic imaging. Further, the vaporization event itself produces a high-contrast photoacoustic imaging signal [16, 25]. The resulting gas microbubble provides sustained ultrasound contrast, enabling dual-mode US-PA imaging from a single agent [26, 52, 53].

Optical absorbers localize within the nanodroplet shell rather than the core. The PFC core is both hydrophobic and lipophobic and therefore rejects most other materials. The shell material must be amphiphilic to emulsify the PFC core, creating a configuration that favors absorber retention in the shell. Even after vaporization, hydrophobic absorbers remain within the shell material, allowing repeated laser pulses to sustain photoacoustic contrast over time [37].

ODV requires sufficient optical penetration to reach target depths, and laser fluence must be carefully controlled to remain within ANSI safety limits and avoid thermal damage to surrounding tissue [25, 54]. Absorber selection directly affects both constraints: NIR-absorbing nanostructures exploit the tissue optical window for deeper penetration, while effective photothermal converters reduce the fluence required for vaporization.

### Combined optical-acoustic droplet vaporization

Combined optical-acoustic droplet vaporization combines laser and ultrasound stimulation to enable vaporization below the individual ODV and ADV thresholds and to enhance spatiotemporal control [34, 55–57]. Mechanistically, ultrasound rarefaction lowers the pressure surrounding the droplet, reducing the fluence threshold required for ODV. Conversely, photothermal heating from the laser reduces the acoustic pressure required for ADV [34]. Together, these effects lower the nucleation barrier to vaporization, and synchronizing the laser pulse with the rarefaction phase can further reduce the vaporization threshold.

The combined modality enhances both vaporization efficiency and therapeutic bioeffects. Synchronized optical-acoustic vaporization increases inertial cavitation activity and sonoporation rates, improving intracellular delivery of therapeutic agents while maintaining low cell mortality [58]. Temporal coordination, such as delivering laser pulses at peak rarefaction, extends bubble lifetime and maximizes mechanical effects [59]. Cavitation intensity and bubble dynamics can be further modulated by adjusting laser PRF and ultrasound pressure.

Spatially localized optical-acoustic vaporization uses the focal confinement of ultrasound to limit the optical vaporization region, even with a relatively broad laser beam. This approach improves targeting accuracy and reduces off-target thermal exposure. However, successful implementation requires a precise sequence and comprehensive characterization of the combined parameters to prevent unintended thermal or mechanical damage. Current research focuses on optimizing synchronization protocols, exploring absorber materials suited to dual-modality vaporization, and assessing long-term safety under clinically relevant conditions.

## Imaging applications of phase-change perfluorocarbon nanodroplets

### Contrast-enhanced ultrasound (CEUS) imaging

**Fig. 3:**
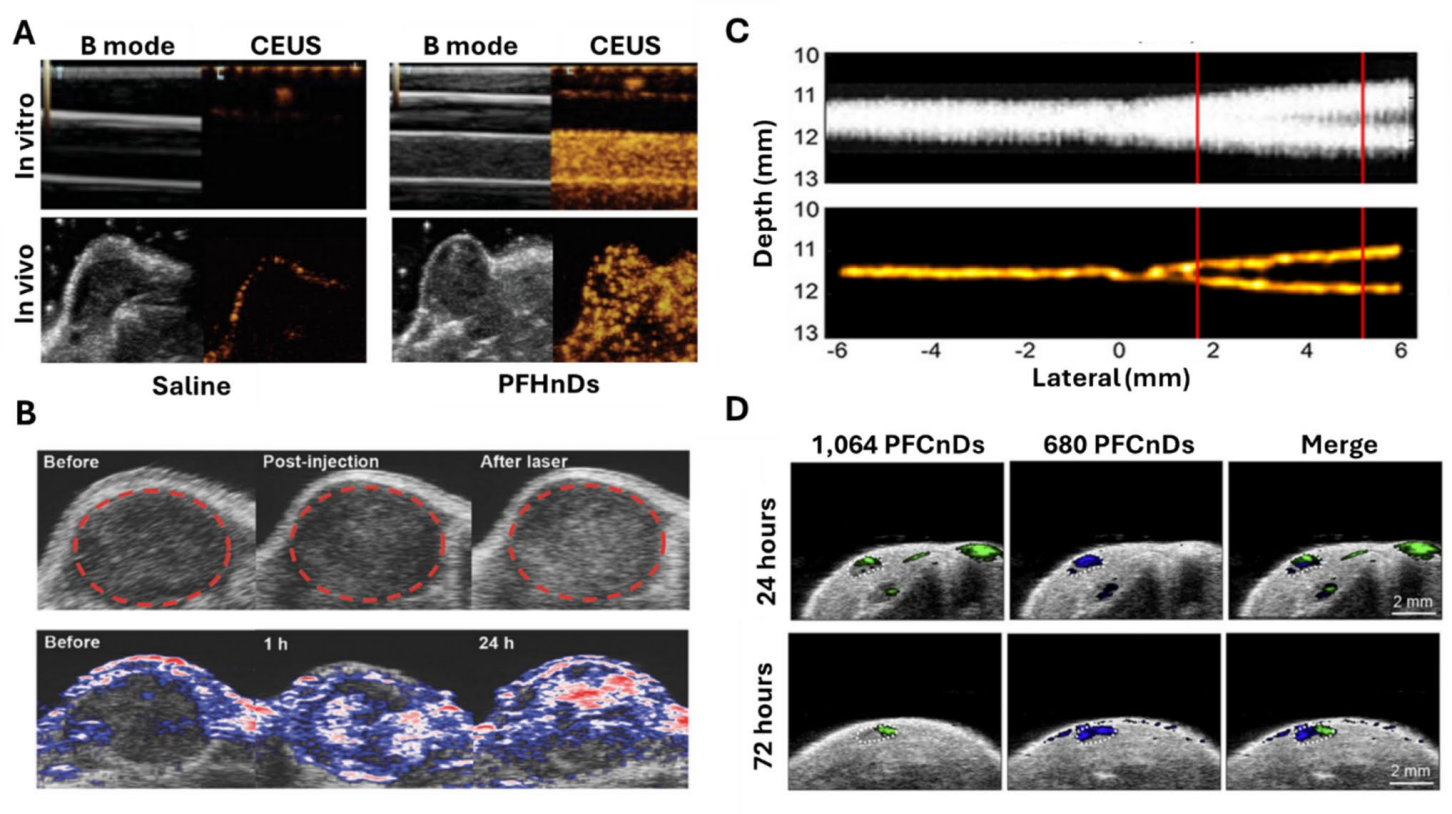
Imaging applications of PFCnDs. **A** Contrast-enhanced ultrasound (CEUS) with PFCnDs: representative B-mode and CEUS images demonstrate robust ultrasound contrast generation by PFCnDs in vitro and in vivo. Reproduced with permission from [67]. **B** Extravascular tumor imaging with PFCnDs: the nanoscale size of PFCnDs facilitates extravasation from tumor vasculature and accumulation within tumor tissue, enabling ultrasound imaging of extravascular tumor regions following vaporization. Reproduced with permission from [75]. **C** Super-resolution ultrasound imaging with PFCnDs: Blinking PFCnD signals yield sparse localizations that can be temporally accumulated to reconstruct microvascular structures beyond the conventional diffraction limit. Reproduced with permission from [83] Copyright 2021 American Chemical Society. **D** Multiplexed lymphatic imaging with PFCnDs: color-coded PFCnD formulations enable multiplexed ultrasound and photoacoustic imaging to distinguish multiple agents while supporting longitudinal visualization of lymphatic structures and drainage pathways. Reproduced with permission from [80]

Phase-change PFCnDs enable contrast-enhanced ultrasound (CEUS) imaging with on-demand vaporization, providing perfusion and vascular mapping capabilities that complement conventional microbubble agents (Fig. 3A) [7, 64, 67]. Upon acoustic droplet vaporization, the resulting microbubbles produce strong backscatter, allowing real-time visualization of blood flow and tissue perfusion. Unlike always-on microbubbles, PFCnDs remain deactivated until triggered, reducing background signal and enabling a selective imaging plane [43, 68–70]. This property has been exploited to assess tumor vascularity, with localized vaporization revealing intratumoral perfusion patterns that yield improved signal-to-noise ratios compared with passive agents [71]. Molecular imaging applications further extend this capability by targeting droplets to specific biomarkers. Ligand-conjugated PFCnDs have been used to image endothelial markers such as VEGFR2 in tumor vasculature, where vaporization yields localized contrast enhancement that correlates with receptor expression levels [30, 44, 72]. The submicrometer size of nanodroplets facilitates binding to vascular epitopes without inducing flow artifacts, and their switchable nature allows repeated imaging sessions with minimal background signal [73].

PFCnD-based CEUS has demonstrated utility in hepatic lesion characterization and myocardial perfusion assessment in preclinical models. The ability to repeatedly vaporize droplets at different time points enables dynamic contrast studies that track perfusion changes during therapeutic interventions, providing real-time treatment monitoring capabilities not achievable with conventional contrast agents [7, 71].

### Extravascular and lymphatic imaging

The submicrometer dimensions of PFCnDs enable them to extravasate from leaky tumor vasculature and penetrate the interstitial space, a capability largely inaccessible to conventional microbubbles (Fig. 3B) [35, 74, 75]. Following systemic administration, droplets accumulate in the tumor via the enhanced permeability and retention (EPR) effect, where they can be vaporized to visualize extravascular structures and monitor drug delivery [12, 43, 76–78]. This approach has been demonstrated in tumor models, where vaporized droplets reveal tumor margins and heterogeneity with high spatial contrast and resolution.

Lymphatic imaging represents another key application. Nanodroplets injected interstitially are taken up by lymphatic vessels and transported to sentinel lymph nodes, where vaporization enables non-invasive mapping of lymphatic drainage pathways and nodal metastasis [79, 80]. The ability to control vaporization timing ensures that only droplets within the node are vaporized, minimizing artifacts from surrounding tissue and allowing quantitative assessment of lymphatic function.

### Super-resolution imaging and localization-based imaging

Phase-change PFCnDs enable super-resolution ultrasound imaging by providing sparse, controllable vaporization events that enable localization at a resolution finer than ultrasound’s physical diffraction limit. Unlike microbubbles, which provide continuous ultrasound contrast, nanodroplets remain inactive until triggered, allowing precise control over the number of contrast sources per frame. Low-amplitude ultrasound pulses vaporize a small fraction of droplets, and each generated microbubble can be localized with high precision. By accumulating localizations from multiple microbubbles over time, it is possible to reconstruct images with subwavelength resolution (Fig. 3C) [64, 81–83]. This approach has resolved microvascular structures down to tens of micrometers in vivo, revealing detailed angioarchitecture in tumor and brain models.

Higher-boiling-point perfluorocarbons such as perfluorohexane (boiling point 56 °C) extend this capability by enabling repeated vaporization and recondensation cycles. Transient microbubbles persist for tens to hundreds of milliseconds before recondensing [37]. Because this recondensation process is stochastic, only a sparse subset of nanodroplet transitions from gas to liquid at any given time, producing temporal separation without requiring spatial sparsity. High-frame-rate ultrasound can capture individual recondensation events, and the precise location of each nanodroplet can be estimated by fitting the ultrasound signal to a two-dimensional Gaussian point spread function [66]. This localization precision achieves subwavelength accuracy nearly an order of magnitude better than conventional ultrasound imaging, enabling high-resolution molecular imaging deep in tissue.

The blinking strategy offers several advantages: background-free imaging without requiring complex filtering, increased localization events per particle, and compatibility with extravascular and lymphatic imaging. However, challenges remain in achieving consistent blinking behavior across heterogeneous droplet populations. Also, the stochastic nature of recondensation necessitates careful adjustment of imaging parameters to balance localization density and acquisition speed. Acquisition speed is a key consideration for clinical translation, where long imaging times are impractical for patient care. Current research seeks to refine PFC composition, shell properties, and absorber concentration to enhance blinking reproducibility and extend super-resolution capabilities to deeper tissue.

### Multiplexed imaging

Multiplexed imaging distinguishes multiple droplet populations within the same field of view by exploiting differences in their vaporization spectra or optical properties. For example, droplets loaded with optical absorbers having distinct absorbance spectra can be selectively vaporized using wavelength-specific laser pulses, enabling spectral unmixing and simultaneous visualization of multiple molecular targets (Fig. 3D) [80]. Alternatively, droplets with different PFC cores with different boiling points exhibit varying acoustic thresholds, allowing sequential vaporization at increasing ultrasound pressures to differentiate populations [62]. This multiplexing capability is particularly valuable for studying complex biological processes, such as tracking multiple cell types or monitoring concurrent drug delivery and therapeutic response.

## Therapeutic application of phase-change perfluorocarbon nanodroplets

PFCnDs utilize phase-change properties to facilitate drug delivery, biological barrier opening, and mechanically mediated bioeffects with precise spatial and temporal control. Combined with the imaging techniques described above, PFCnDs are also employed in image-guided therapy [84].

### Drug delivery

PFCnDs enable localized drug release by encapsulating therapeutic agents within the PFC core or attaching them to the shell, with controlled release triggered by vaporization-induced shell rupture or bubble oscillation [4, 7]. Chemotherapeutics such as doxorubicin have been co-encapsulated with PFC in lipid-based or PLGA-based nanodroplets, where low-intensity focused ultrasound (LIFU) induces phase change and microbubble collapse, releasing the payload at the target site. The acoustic parameters can be tuned to achieve programmable release profiles; for instance, lipid shells require lower acoustic pressures for vaporization compared to more rigid PLGA shells, allowing sequential vaporization of different nanodroplet formulations for temporal control over drug delivery. This approach enhances therapeutic efficacy while reducing systemic toxicity by concentrating the drug at the tumor site.

**Fig. 4:**
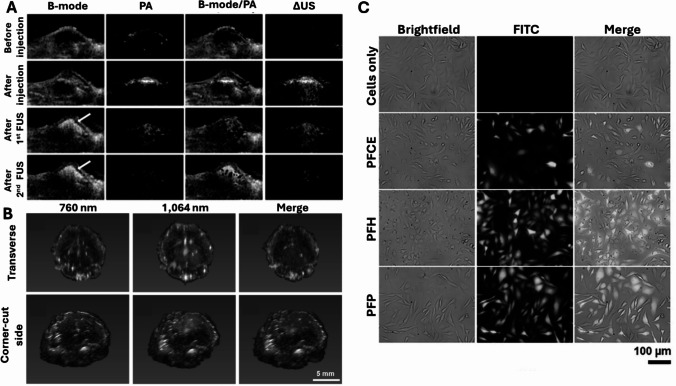
Therapeutic applications of PFCnDs. **A** Drug delivery and release with PFCnDs: US/PA imaging enables visualization of PFCnD accumulation at the target site and confirms localized payload release upon triggering. Reproduced with permission from [85]. **B** BBB opening and intracerebral delivery with PFCnDs: two PFCnD formulations are used, one enables BBB opening via optical vaporization-driven bubble oscillation, while the other demonstrates delivery of therapeutics across the opened BBB. Reproduced with permission from [65]. **C** Sonoporation-enabled transfection: PFCnD vaporization–induced oscillation promotes sonoporation, leading to enhanced cellular transfection (e.g., eGFP expression). Reproduced with permission from [89]

A significant challenge in PFCnD-based drug delivery has been the encapsulation of hydrophilic therapeutics, as the hydrophobic and lipophobic nature of perfluorocarbons prevents direct dissolution of water-soluble drugs. Double-emulsion formulations have been developed to address this limitation. Double-emulsion PFCnD (dePFCnD) possesses an internal hydrophilic core surrounded by a PFC layer, which significantly improves the encapsulation efficiency of hydrophilic therapeutics while maintaining a size comparable to that of single-emulsion PFCnDs (Fig. 4A) [42, 85, 86].

dePFCnDs have also been extended to co-delivery of multiple drugs with distinct physicochemical properties. A modified double-emulsion technique enables dual-drug-loaded nanodroplets (DDDs) to co-encapsulate paclitaxel (lipophilic) and doxorubicin (hydrophilic), supporting combination chemotherapy with synchronized pharmacokinetics [42]. Upon ultrasound triggering, the DDDs released both agents at the tumor site, demonstrating enhanced cytotoxicity and reduced tumor growth compared to passive accumulation via the EPR effect alone. Beyond small-molecule chemotherapeutics, recent work has explored dePFCnDs for precision delivery of biologics such as mRNA and ribonucleoproteins, further expanding the therapeutic repertoire. Also, PFCnDs have been loaded with siRNA, thrombolytic agents (e.g., thrombin), and other macromolecular payloads, exploiting the mechanical forces generated during vaporization to facilitate intracellular uptake via sonoporation [87].

Monitoring droplet distribution with US or PA imaging before drug release provides real-time feedback, ensuring that drug release occurs only within the intended region. However, challenges remain in optimizing loading efficiency, maintaining payload stability during prolonged circulation, and achieving complete release upon triggering without excessive tissue damage. The interplay among shell composition, PFC volatility, and acoustic parameters must be carefully balanced to ensure vaporization at clinically safe exposure levels while maximizing therapeutic payload delivery.

### Blood–brain barrier opening

Optically vaporized PFCnDs have been investigated as a noninvasive tool for transiently opening the blood–brain barrier (BBB), offering an alternative or complement to microbubble-assisted focused ultrasound (Fig. 4B) [27, 65, 88]. Perfluorohexane nanodroplets (PFHnDs) encapsulated with NIR optical absorbers undergo vaporization upon pulsed laser irradiation, generating mechanical forces that disrupt tight junctions and enable extravasation of therapeutics into brain parenchyma. The extent of BBB opening can be controlled by modulating laser fluence and pulse number [65].

Acoustically vaporized PFCnDs also promote BBB permeability via acoustic droplet vaporization, during which microbubble cavitation applies mechanical stress on endothelial cells [27]. In vitro studies using brain endothelial cell monolayers demonstrate that nanodroplets combined with ultrasound increase paracellular permeability, with the effect dependent on acoustic pressure and droplet concentration. The submicrometer size of PFCnDs may facilitate accumulation in tumor vasculature or at sites of inflammation, potentially enabling targeted opening of the BBB in pathological regions while sparing healthy brain tissue. Repeated vaporization cycles of PFHnDs allow multiple opportunities for BBB interaction, a unique advantage over single-use microbubbles. However, optimization of laser parameters, acoustic pressure, and droplet dose is critical to minimize tissue damage, hemorrhage, and inflammation, which have been observed at excessive fluences.

### Mechanically driven bioeffects

The mechanical forces generated during PFCnD vaporization and subsequent bubble dynamics can drive therapeutic bioeffects beyond drug delivery, including sonoporation, thrombolysis, and immune modulation (Fig. 4C) [34, 45, 89]. Sonoporation—the transient permeabilization of cell membranes via cavitation—has been achieved using repeated vaporization, in which mechanical stress from bubble oscillation creates nanoscale pores in the plasma membrane, enhancing the uptake of macromolecules without inducing significant cell death [34, 57]. Unlike inertial cavitation, which requires high pressures and risks cell lysis, repeated vaporization at moderate pressures (0.5–1.5 MPa) can sustain sonoporation over thousands of cycles, providing a controlled method for intracellular delivery. Synchronizing laser pulses with the rarefaction phase of ultrasound further enhances vaporization efficiency and reduces the acoustic pressure required, enabling effective sonoporation with lower energy deposition [57].

Beyond cellular permeabilization, PFCnDs have been explored for sonothrombolysis, where vaporization-induced microstreaming and shear stresses disrupt thrombi. The ability to repeatedly vaporize droplets enables sustained mechanical agitation of clots, potentially improving recanalization efficacy over single-pulse microbubble approaches. Mechanically mediated bioeffects also extend to immune modulation; cavitation can induce local inflammation and antigen release, which may be leveraged to enhance immunotherapy responses, though this remains largely speculative and requires further investigation. The safety profile of these mechanical effects is paramount, as excessive cavitation risks vascular damage, hemorrhage, and tissue injury. Therefore, careful characterization of the parameter space—balancing therapeutic efficacy with bioeffect mitigation—is essential for clinical translation of PFCnD-based mechanical therapies.

## Challenges and translational outlook

Despite promising preclinical advances, the clinical translation of PFCnDs faces significant challenges in formulation, manufacturing, and safety. While technical optimization continues, deeper structural barriers, such as manufacturing standardization, robust vaporization behavior, and systematic safety assessment under clinically heterogeneous conditions, require deliberate attention to avoid translation failures.

### Formulation and manufacturing challenges

A primary challenge in clinical translation is the control of PFCnD physical characteristics, particularly size distribution, yield, and batch-to-batch reproducibility, under manufacturing conditions relevant to clinical use [10]. Most laboratory-scale fabrication approaches, such as tip sonication, produce polydisperse populations with coefficients of variation often exceeding 20%, resulting in broad distributions of vaporization thresholds [20]. This polydispersity complicates the interpretation of vaporization behavior, hinders precise dosing, and can result in heterogeneous bioeffects when identical exposure parameters are applied across a droplet population. Achieving submicrometer, narrowly distributed droplet sizes is critical for predictable vaporization, consistent biodistribution, and reliable extravasation into target tissues [8].

Yield and scalability constitute further obstacles. Traditional batch emulsification typically yields only a few grams of nanodroplets per day, which is insufficient for large-animal studies or clinical trials. Continuous-flow microfluidic systems have substantially increased production rates to tens of grams per day, while also providing improved control over droplet size, PFC loading, and polydispersity [21, 90]. However, these systems introduce new engineering challenges, including channel fouling, long-term operational stability, and the need for integrated downstream purification. Labor-intensive centrifugation steps used for lab-scale purification are impractical at the clinical scale and must be replaced by scalable processes such as tangential-flow filtration or in-line size-based separation [22].

Biosafety and stability are critical considerations. PFCnDs must remain stable through storage, shipping, and clinical preparation without unintended phase change or aggregation that could alter size or vaporization behavior [8]. Low-boiling-point PFCs enhance sensitivity but increase the risk of spontaneous vaporization or gas evolution over time [9]. Shell materials and excipients must be biocompatible, non-immunogenic, compatible with existing administration routes, and support sterilization and long-term storage without significant changes in droplet characteristics [17, 24]. Comprehensive physicochemical characterization, including assessment of size distribution, surface charge, PFC content, and stability under clinically relevant temperature and pressure conditions, is essential for clinical translation.

### In vivo fate and safety considerations

The in vivo behavior of PFCnDs, including biodistribution, clearance, and long-term biocompatibility, remains incompletely characterized [13]. Perfluorooctyl bromide (PFOB)-based nanodroplets exhibit a few hours' blood half-life in rodents with complete organ clearance over several days, suggesting that elimination is feasible but depends on both core and shell design [74]. Different shell materials—lipid versus polymer—produce distinct pharmacokinetic and organ-uptake profiles, altering residence times in the liver, spleen, and lungs and potentially modulating immune responses. Detailed studies of reticuloendothelial system uptake, PFC redistribution, and excretion routes are needed to assess cumulative exposure and long-term safety, especially for repeated dosing paradigms envisioned in chronic imaging or therapy.

Vaporization safety poses a parallel concern. Unintended droplet-to-bubble conversion within the circulation, particularly at off-target sites or under non-optimized ultrasound or laser exposure, risks embolic phenomena and microvascular obstruction [45]. Excessive acoustic pressures or laser fluences can induce inertial cavitation and vascular damage, including endothelial disruption, hemorrhage, and inflammatory responses, especially in sensitive tissues such as the brain during blood–brain barrier opening [91]. The narrow margin between effective vaporization and adverse bioeffects underscores the need for precisely defined exposure windows, robust dosimetry, and real-time monitoring of vaporization and cavitation activity [32].

For translational contexts, vaporization safety should be interpreted against quantitative regulatory and consensus-reference limits. For ADV, the FDA-defined diagnostic ultrasound output framework commonly cites derated limits of mechanical index (MI) ≤ 1.9, with a spatial-peak temporal-average intensity I_SPTA_ ≤ 720 mW/cm^2^, with more restrictive limits for ophthalmic applications (e.g., MI ≤ 0.23) [92]. Because the mechanical index is defined as MI = PNP (MPa)/$\sqrt{f (MHz)}$ (f: frequency), ADV thresholds should not be interpreted solely in terms of PNP; their clinical relevance depends on both pressure and frequency. In clinical contrast-enhanced ultrasound with FDA-approved microbubble agents, practical operating settings are typically more conservative than the general device limits, with product labeling commonly recommending low-MI imaging, such as MI ≤ 0.8 for echocardiographic use and MI ≤ 0.4 for some contrast-specific abdominal applications, because increasing MI accelerates bubble destruction and may increase cavitation-related bioeffects. These FDA values are derated quantities rather than direct in situ measurements; the acoustic pressure delivered to the target can differ substantially from free-field or water-path measurements due to tissue attenuation (approximately 0.5–0.7 dB/cm/MHz), aberration, focusing geometry, and target depth. For ODV, safety comparison should likewise be made to the applicable ANSI maximum permissible exposure (MPE), which depends on wavelength, pulse duration, repetition rate, exposure geometry, and whether ocular or skin exposure is limiting, rather than to a single universal fluence threshold [54]. Accordingly, surface-incident fluence should not be treated as equivalent to in situ optical exposure at depth, which is further shaped by absorption, scattering, beam profile, and propagation distance in tissue. For phase-change PFC nanodroplets, however, analogous exposure definitions and safety benchmarks remain less standardized than for approved microbubble agents. This gap highlights the need for a nanodroplet-specific safety framework that defines clinically relevant exposure metrics, acceptable operating margins, vaporization endpoints, and cavitation-related risk thresholds for different translational use cases.

Safety evaluation must therefore encompass not only conventional toxicology but also mechanobiological endpoints, including vascular integrity, tissue injury, and immune activation under repeated vaporization cycles [93].

### Vaporization reproducibility and standardized metrics

Vaporization thresholds serve as a foundation for predicting clinical behavior, yet direct numerical comparisons across studies are often impossible due to variability in experimental methods. Key ADV factors, including peak negative pressure, pulse duration, PRF (1–100 Hz), and frequency (1–15 MHz), vary widely without standardization [15, 32, 33]. For ODV, key factors (e.g. laser fluence, beam profile, tissue depth) are either omitted entirely, or when reported, exhibit significant variations across studies regarding wavelength (532–1064 nm), absorber concentration (20–100-fold variation), and pulse duration. Vaporization metrics also differ, including acoustic backscatter intensity, photoacoustic signal, and passive acoustic signal.

To facilitate reproducibility, studies should report all relevant exposure parameters and distinguish whether the values apply to the output at the transducer or tissue surface, or to the estimated in situ exposure at the target. For ultrasound, this includes reporting whether values are measured in water, derated, or estimated in situ, together with frequency, pulse structure, depth, and focusing conditions. For optical triggering, reporting should include wavelength, pulse duration, repetition rate, spot size, beam profile, surface fluence, and the basis used to estimate in situ fluence at depth.

The broader ultrasound field has established standardized reporting practices, but no analogous framework exists for PFCnD vaporization. Recent initiatives have begun systematizing reporting, but field-wide adoption remains inconsistent. Regulatory agencies require dose–response relationships linking exposure to safety and efficacy endpoints; foundational work is largely absent for PFCnDs. To achieve reproducible and interpretable results, studies must adopt robust reporting practices; to facilitate this, the parameters outlined above are offered as a foundational baseline.

## Conclusion

PFCnDs serve as phase-changeable, multimodal agents that expand the capabilities of conventional ultrasound contrast beyond the vascular compartment. Their submicrometer size, prolonged circulation, and switchable contrast generation enable precise, on-demand imaging and therapy. Formulation flexibility supports the integration of targeting ligands, optical absorbers, and therapeutic payloads. The thermodynamic and kinetic framework governing acoustic, optical, and combined optical-acoustic vaporization provides a unified basis for understanding and optimizing droplet behavior across diverse applications.

Successful translation from preclinical promise to clinical application requires rigorous attention to manufacturing scalability, in vivo fate, and safety profiling. Addressing challenges such as hydrophilic drug loading, size heterogeneity, and unintended vaporization requires advanced formulation design and continuous-flow production under quality-by-design principles. Standardized metrics for vaporization thresholds and bioeffect characterization are essential to harmonize research efforts and guide regulatory approval. With sustained interdisciplinary effort and robust clinical validation, PFCnDs are poised to become next-generation theranostic platforms, providing image-guided, site-selective therapy that complements current ultrasound-mediated interventions.
